# Are polypropylene mesh implants associated with systemic autoimmune inflammatory syndromes? A systematic review

**DOI:** 10.1007/s10029-021-02553-y

**Published:** 2022-01-12

**Authors:** C. R. Kowalik, S. E. Zwolsman, A. Malekzadeh, R. M. H. Roumen, W. A. R. Zwaans, J. W. P. R. Roovers

**Affiliations:** 1 Department of Gynecology, Amsterdam University Medical Centre, Room H4-262, PO Box 22660, 1100 DD Amsterdam, The Netherlands; 2 Department of Surgery, Máxima Medical Centre, Veldhoven/Eindhoven, The Netherlands; 3 Research Consortium Mesh, Utrecht, The Netherlands; 4 NUTRIM School of Nutrition and Translational Research in Metabolism, Maastricht University Medical Centre, Maastricht, The Netherlands; 5 Bergman Clinics, Amsterdam, The Netherlands

**Keywords:** Pelvic organ prolapse, Polypropyleen, Mesh, Inguinal hernia, Systemic autoimmune disorders, Implant

## Abstract

**Purpose:**

The surgical implantation of polypropylene (PP) meshes has been linked to the occurrence of systemic autoimmune disorders. We performed a systematic review to determine whether PP implants for inguinal, ventral hernia or pelvic floor surgery are associated with the development of systemic autoimmune syndromes.

**Methods:**

We searched Embase, Medline, Web of Science, Scopus, Cochrane library, clinicaltrialsregister.eu, clinicaltrails.gov and WHO-ICTR platform. Last search was performed on November 24th 2021. All types of studies reporting systemic inflammatory/autoimmune response in patients having a PP implant for either pelvic floor surgery, ventral or inguinal hernia repair were included. Animal studies, case reports and articles without full text were excluded. We intended to perform a meta-analysis. The quality of evidence was assessed with the Newcastle–Ottawa Scale. This study was registered at Prospero (CRD42020220705).

**Results:**

Of 2137 records identified, 4 were eligible. Two retrospective matched cohort studies focused on mesh surgery for vaginal prolapse or inguinal hernia compared to hysterectomy and colonoscopy, respectively. One cohort study compared the incidence of systemic conditions in women having urinary incontinence surgery with and without mesh. These reports had a low risk of bias. A meta-analysis showed no association when comparing systemic disease between mesh and control groups. Calculated risk ratio was 0.9 (95% CI 0.82–0.98). The fourth study was a case series with a high risk of bias, with a sample of 714 patients with systemic disease, 40 of whom had PP mesh implanted.

**Conclusion:**

There is no evidence to suggest a causal relationship between being implanted with a PP mesh and the occurrence of autoimmune disorders.

**Supplementary Information:**

The online version contains supplementary material available at 10.1007/s10029-021-02553-y.

## Introduction

In patients with pelvic organ prolapse (POP) or inguinal hernia, surgical outcome with native tissue has a high risk of recurrence. The introduction of polypropylene (PP) implants to surgically repair connective tissue defects has resulted in improved surgical outcome [1, 2]. These implants have been used since the 1960s for inguinal and ventral hernia repair and since the 90s for stress urinary incontinence (SUI) and POP repair [3–6]. Although PP implants have been proven to decrease the recurrence risk, the risk of mesh-related complications has to be weighed against the benefits.

Well-known mesh-related complications include nerve entrapment, mesh erosion, mesh exposure and pain [7, 8]. Whether the occurrence of systemic inflammatory symptoms can also be considered a mesh-related complication is still under debate. It has been postulated that PP can cause a systemic autoimmune inflammatory disorder, as has been described in women with silicone breast implants, called autoimmune/inflammatory syndrome induced by adjuvants (ASIA) [9]. The rationale behind this hypothesis is that the local inflammatory reaction after mesh insertion might result in a systemic upregulation of inflammatory mediators [10]. If PP would prove to be an adjuvant for the development of systematic inflammatory response symptoms, this would have huge implications for the treatment of patients with symptoms of systemic immune disease, as it would imply that only a complete mesh removal could result in symptom reduction. Such surgery is invasive, can be technically challenging and would therefore only be acceptable if the indication is indisputable.

The objective of this systematic review is to study if there is an association with PP implants for inguinal and ventral hernia repair or pelvic floor surgery and the development of systemic autoimmune syndromes. All types of studies reporting the outcome of developing systemic autoimmune syndrome(s) in patients having a PP mesh implant for SUI, POP, ventral or inguinal hernia were systematically reviewed.

## Materials and methods

This review was conducted according to PRISMA guidelines [11]. The protocol was previously registered and published in Prospero (https://www.crd.york.ac.uk/prospero; Registration number: CRD42020220705).

A narrative review in Dutch describing the systemic effect of PP implants in Urogynecology has been published previously [12].

### Eligibility criteria

This study was set up according to the PICO framework for the domain of harm.

Inclusion criteria were experimental, prospective, cross-sectional and observational studies (case–control studies, cohort studies, case series) reporting evolvement of systematic inflammatory or autoimmune diseases after PP implantation. We required full journal publication, with the exception of online clinical trial results, summaries of otherwise unpublished clinical trials and abstracts with sufficient data for analysis.

Studies describing PP implants not intended for POP, SUI, ventral hernia or inguinal hernia were excluded. Other exclusion criteria were case reports and articles describing data obtained from animal studies. Neither language restriction nor time limitations were imposed.

Patients > 18 years of age and having a PP mesh implant for either POP, SUI, ventral hernia or inguinal hernia were considered. The outcome was a systemic inflammatory or autoimmune response.

### Search strategy

A systematic search strategy was developed to identify published studies on Embase (Ovid SP platform), Medline (Ovid SP platform), Web of Science, Scopus and Cochrane library. Furthermore, clinicaltrialsregister.eu, clinicaltrails.gov and WHO-ICTR platform were searched to include unpublished trial reports. Lastly, upon final inclusion of relevant studies a snowball method (forward and backward reference checking) was performed on Google Scholar and Microsoft Academics to avoid missing relevant papers.

The searches were performed and concluded on November 24th 2021. Subsequently, forward and backward searches were performed on November 24th 2021. Three different search blocks containing a combination of Mesh/Emtree and free text combinations were applied as follows (full search strategy can be found in Appendix A):

1. (pelvic organ prolapse or uterine prolapse or Hernia, Ventral or Hernia, Inguinal or Urinary Incontinence, Stress or Cystocele or Hernia, Abdominal or Rectocele or Herniorrhaphy)

AND

2. (Polypropylenes or Surgical Mesh)

AND

3. (Autoimmunity or autoimmune diseases or systemic inflammatory response syndrome or Inflammation or Foreign-Body Reaction)

### Selection of articles

Two reviewers (SZ, CK) independently did the screening and selection of the studies. Before starting the selection of articles, the reviewers had a meeting to discuss the eligibility criteria. For selecting eligible studies, Rayyan QCRI (https://rayyan.qcri.org) was used.[13]

**Figure 1.**
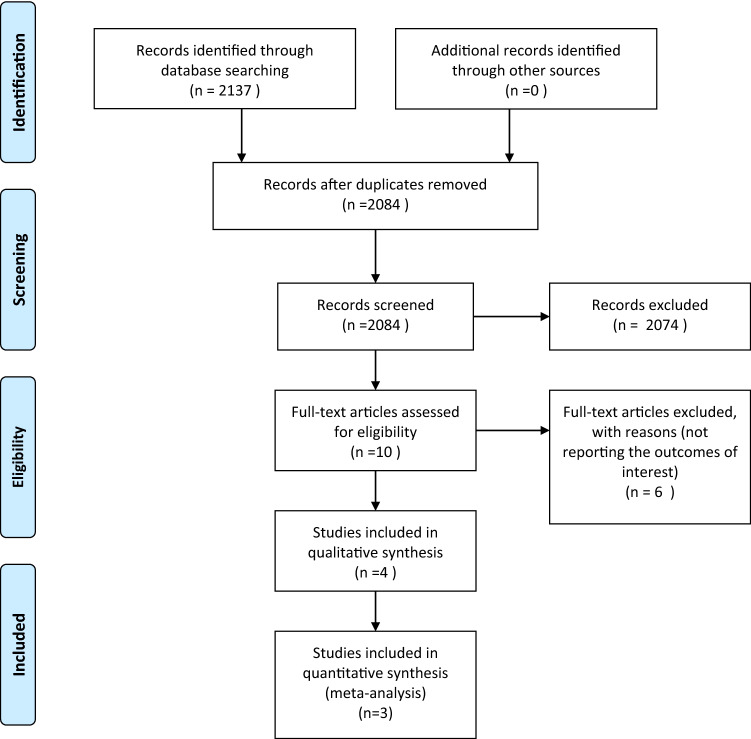
Flow chart of study inclusion.

Both reviewers screened all articles, first titles, then abstracts and lastly full texts. A flowchart of study selection according to the PRISMA statement provides insight into the screening process (see Fig. 1).

### Data extraction (selection and coding)

Data extraction was done by CK using a predefined form, that included author, country, year of publication, journal, publication type, aim, study type, source of patients, primary outcome, follow-up time, mean age, number of patients included, gender, eligibility criteria and results. Subsequently, a second reviewer (SZ) checked extracted data.

### Risk of bias (quality) assessment

Risk of bias was assessed using the Newcastle–Ottawa Scale at study level [14]. For each included study, the appropriate design scale was used. Two reviewers (SZ, WZ) independently assessed the risk of bias of the included studies using this validated tool. A description of risk of bias was done, as suggested by the scale developers. The quality of each study, including selective reporting within a study, will be weighted in the conclusion of this review.

### Strategy for data synthesis

Data have been summarized narratively by outcomes that were described in the particular study articles. If more than one comparative study was found, a meta-analysis was performed and *I*^2^ presented.

### Analysis of subgroups or subsets

Further subgroup analyses will be performed if appropriate.

## Results

A total of four studies have been included in this review. The search of Embase (Ovid SP platform), Medline (Ovid SP platform), Web of Science, Scopus and Cochrane library and clinicaltrialsregister.eu, clinicaltrails.gov and WHO-ICTR platform provided 2137 citations. After removing duplicates 2084 records have been screened. Screening on title and abstract resulted in ten full-text articles that were assessed for eligibility. Six of these did not meet the inclusion criteria. Finally, four studies were included in the present review (see Fig. 1).

### Critical appraisal

To determine the quality of the included studies, the Newcastle Ottawa Scale was used. For both studies of Chughtai [15, 16], the risk of bias is low: the selection of patients was considered representative, the cohort selection was done appropriately, surgical records seemed appropriate for selecting patients and outcomes were not present at start of the study. Comparability of cohorts was assessed. The outcomes were derived through record linkage, which imposes medium risk of bias. Follow-up for both the exposed cohort as well as controls was two years and considered appropriate.

The risk of bias for the study of Muller [17] was considered low since a large number of patients were selected from a cohort of women who underwent SUI surgery with (intervention) or without (controls) mesh. The minimal follow-up duration of 5 years should be adequate and the study corrected for significant confounding factors, including age, ethnicity and pre-existing comorbidities. The study of Cohen Tervaert [9] has low risk of bias considering the selection of patients with autoimmune disease, but patients were selected from a cohort with known autoimmune symptoms, and therefore, the outcome was present at start of the study. A non-exposed cohort was lacking. There was no description of ascertainment of exposure. Comparability of cohorts based on neither design nor analysis was described. Outcome assessment was done by record linkage. Follow-up was not performed (see Table 1).

**Table 1:**
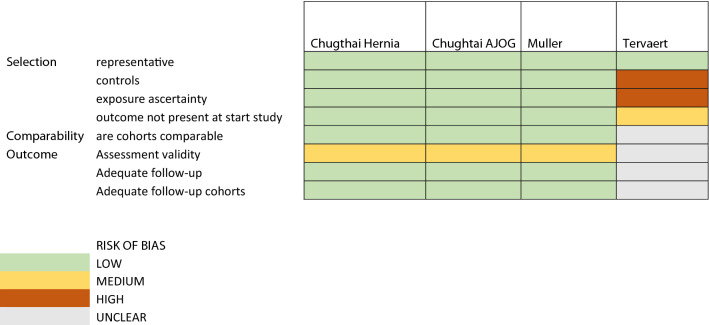
Risk of bias

### Study characteristics

Two of the selected studies were retrospective cohort studies with matched controls and were performed in the USA, by Chughtai et al. [15, 16]. One study was a national cohort study that has been carried out in the United Kingdom by Muller et al. [17].

Cohen Tervaert performed the fourth study in the Netherlands, Canada and Belgium. This was a case series [9]. The summary of included studies is shown in Table 2.

**Table 2:** Characteristics of the included studies

Authors	No. of patients	Inclusion Criteria	Age (years)	FU time	Results
Chugtai et al. [15]	*N* = 12,716 mesh hernia *N* = 25,432 Colonoscopy	Inclusion: Patients undergoing PP mesh inguinal hernia repair For control: patients undergoing colonoscopy	Cohort: 57.8 (12.8)* Control: 57.8 (12.8)*	At 6 months, 1 year, 2 years and during the entire FU period (average was 6 years)	Cohort: 188 patients (1.5%) had developed SAID at the end of follow-up Control: 413 patients (1.6%) had developed autoimmune disease at the end of follow-up. Result: Adjusted OR 0.91; 95% CI (0.76–1.09). No association was found between hernia mesh repair and the development of SAID at 6-month, 1-year and 2-year FU
Chugtai et al. [15]	*N* = 1507 mesh *N* = 3014 Colonoscopy *N* = 1375 Hysterectomy	Inclusion: Women undergoing mesh POP-repair For control: Women undergoing screening colonoscopy (non-surgical cohort) or hysterectomy for benign gynecologic or urogynecologic indications	Cohort: 60.4 (11.5)* Control: 60.4 (11.6)*	At 6 months, 1 year, 2 years and during the entire FU period (average was 6 years)	Mesh vs. Colonoscopy: Mesh cohort: 2.8% of patients developed SAID. Control: 2.8% of patients SAID. Adjusted OR 0.91; 95% CI (0.62–1.34) Mesh vs. vaginal hysterectomy: Mesh cohort: 2.8% of patients developed autoimmune disease. Control: 3.2% of patients developed SAID. Adjusted OR 0.78; 95% CI (0.48–1.26)
Tervaert [9]	*N* = 40 (18 hernia, 4 TVT and 18 TVM)	Inclusion: Patients presenting to several autoimmune clinics, who had previously implanted polypropylene mesh	49.5 (range 28–75)	Not explicitly described, symptoms of autoimmune disease were recorded at presentation at the clinic, including whether a patient had a PP implant	18 (45%) of patients were diagnosed with autoimmune disease
Muller [17]	*N* = 88 947 mesh surgery *N* = 3389 non-mesh surgery	Inclusion: Women having first time urinary incontinence surgery with mesh For control: women having first urinary incontinence surgery without mesh	Mesh: 53.1 ± 12* Control: 52.2 ± 12*	Mesh group: 8.7 (6.8–8.7)^¥^ years Non-mesh group: 9.9 (7.4–9.9)^¥^ years	Cumulative incidence of autoimmune disease, fibromyalgia or myalgic encephalomyelitis Mesh group: 8.1% at 10 years Non-mesh group: 9% at 10 years

*Mean (SD)

^¥^Median (IQR)

The studies have been conducted between 2008 and 2019. When combining the three cohort studies eligible for meta-analysis, a total of 104,594 matched participants had PP implants, because of inguinal hernia repair, mesh for POP or SUI. These participants were matched with 33,253 controls.

The control groups in the studies of Chughtai et al. [15, 16] were extracted from a cohort of colonoscopy patients and a second cohort of patients with a history of vaginal hysterectomy. All subjects in the cohort studies were individually matched by patient characteristics and comorbidities. The women included in the study of Muller et al. [17] were all women who had SUI surgery either with or without mesh. Patient characteristics, demographic data and comorbidities were comparable.

The study of Cohen Tervaert [9], being a case series, contained a sample of 714 participants, of whom 40 had a PP mesh implant.

The primary outcome of the studies by Chughtai et al. [15, 16] was the development of systemic autoimmune disorders (SAID) at the entire follow-up period. The average follow-up period of both studies by Chughtai et al., was 6 years. SAID was defined in one study as an enumeration of various autoimmune disorders (Grave’s disease, Hashimoto’s thyroiditis, pernicious anaemia, autoimmune haemolytic anaemia, autoimmune thrombocytopenic purpura, amyotrophic lateral sclerosis, multiple sclerosis, Guillain–Barré Syndrome, myasthenia gravis, Goodpasture syndrome, vasculitis, celiac disease, pemphigus vulgaris, systemic lupus erythematosus, systemic sclerosis, Sjogren’s syndrome, dermatomyositis, polymyositis, rheumatoid arthritis, ankylosing spondylitis and fibromyalgia) [16]. Secondary outcomes included development of SAID at 6-month, 1-year and 2-year follow-up time point.

The main outcome measure of the study of Muller et al. [17] was the first post-operative admission with a record of at least one of 29 autoimmune diseases, fibromyalgia or myalgic encephalomyelitis. Inclusion commenced in 2006 and the study was closed after a minimum follow-up of 5 years for all patients, with a maximum follow-up period of 10 years.

The study of Cohen Tervaert reported on symptoms suggestive of a (systemic) autoimmune disease in the presence of a PP mesh [9]. Autoimmune disease in the presence of a PP implant in this study was defined as fulfilment of the criteria for the diagnosis of autoimmune/inflammatory syndrome induced by adjuvants (ASIA), Shoenfeld’s criteria [18]. (Appendix B). Data were collected at presentation to an autoimmune clinic; no further follow-up was described.

### Outcomes

Chughtai et al. performed two retrospective cohort studies with matched controls [15, 16]. In one study subjects were males who had undergone an inguinal hernia repair with mesh, the other study included women with a POP repair with mesh. The source of patients was the New York State Department of Health Statewide Planning and Research Cooperative System (SPARCS) [19].

The male subjects who were included in the mesh for herniorrhaphy study were matched with a control cohort, consisting of patients undergoing colonoscopy. Controls were excluded if they had a history of mesh-related procedures, a diagnosis of colorectal carcinoma within one month of the colonoscopy or a previous diagnosis of SAID.

In total 12,716 men with a history of (mesh) herniorrhaphy were matched with 25,432 patients who had a colonoscopy. SAID was diagnosed in 188 (1.5%) in the mesh group. In the control group, 413 patients (1.6%) had developed SIAD at the end of follow-up. The adjusted OR was 0.91 (95% CI 0.76–1.09). After matching, the authors concluded that inguinal mesh hernia repair was not associated with the development of SAID [16].

The women who have been enrolled in the POP with mesh repair study were matched with two cohorts of controls: a surgical and a non-surgical cohort. Controls were either women with a vaginal hysterectomy in their medical history for benign gynecological or urogynecological conditions (surgical cohort) or women who had an indication for a screening colonoscopy (non-surgical cohort).

Two thousand one hundred two women with a mesh-repair for POP were included. These were matched with 37,298 women in the non-surgical control cohort and 7338 women in the surgical control cohort. This resulted in 1507 women with mesh repair matched with 3014 colonoscopy patients and 1375 women with mesh repair matched with 1375 women with vaginal hysterectomy.

Subjects with a (concurrent) history of autoimmune disease, malignancy, mesh-related procedures or prior pelvic floor surgery were excluded. An additional exclusion criterion for the non-surgical cohort was inflammatory bowel disease. In the surgical control cohort women with endometrial hyperplasia with atypia, abnormal vaginal bleeding or benign ovarian pathology were excluded.

In total SAID was diagnosed in 59 women (2.8%) after prolapse with mesh repair, in 1060 women (2.8%) after colonoscopy and in 235 women (3.2%) who had a history of vaginal hysterectomy.

After individual matching by demographics, date of the procedure and comorbidities, no increased risk of developing SAID after a mesh implantation for POP was found. The adjusted OR was 0.91 (95% CI 0.62–1.34) when comparing to the colonoscopy group and 0.78 (95% CI 0.48–1.26) when comparing to the vaginal hysterectomy group.

Muller et al. [17] performed a national cohort study to compare the incidence of SAID in women having SUI surgery with and without mesh. Patients who had SUI surgery in the English NHS between 2006 and 2013 were included from an administrative database called the Hospital Episode Statistics.

Women were excluded if they had a record of SUI surgery in the previous 3 years or had a history of autoimmune disease, fibromyalgia or myalgic encephalomyelitis within this timeframe.

In total 88,947 women with mesh surgery and 3389 women without mesh surgery for SUI were included. The cumulative incidence of autoimmune disease, fibromyalgia or myalgic encephalomyelitis was 8.1% (95% CI 7.9–8.3%) in the mesh cohort and 9.0% (95% CI 8.0–10.1%) in the control group. The adjusted HR was 0.89 (95% CI 0.79–1.01; *p* = 0.07).

This study did not demonstrate an increased risk of systemic disease after mesh implantation for SUI.

Finally, we included the study of Cohen Tervaert [9]. This study described 40 patients with systemic complaints in the presence of a PP mesh implant, who were selected out of a cohort of 714 patients who presented to the authors’ autoimmune clinic. Patients were classified as suffering from autoimmune/inflammatory syndrome induced by adjuvants (ASIA syndrome) when they fulfilled Shoenfeld’s criteria (Appendix B) [18]

The author described that in 24 out of 40 of the included patients their symptoms started within 1 year after mesh implantation. Ten out of 40 subjects developed ASIA between 1 and 3 years after the implantation of PP, and in 6 patients these symptoms developed later than 3 year after PP implantation. Eighteen out of 40 patients were diagnosed with an International Classification of Diseases (ICD) coded autoimmune disease.

### Synthesis of results

**Fig. 2:**
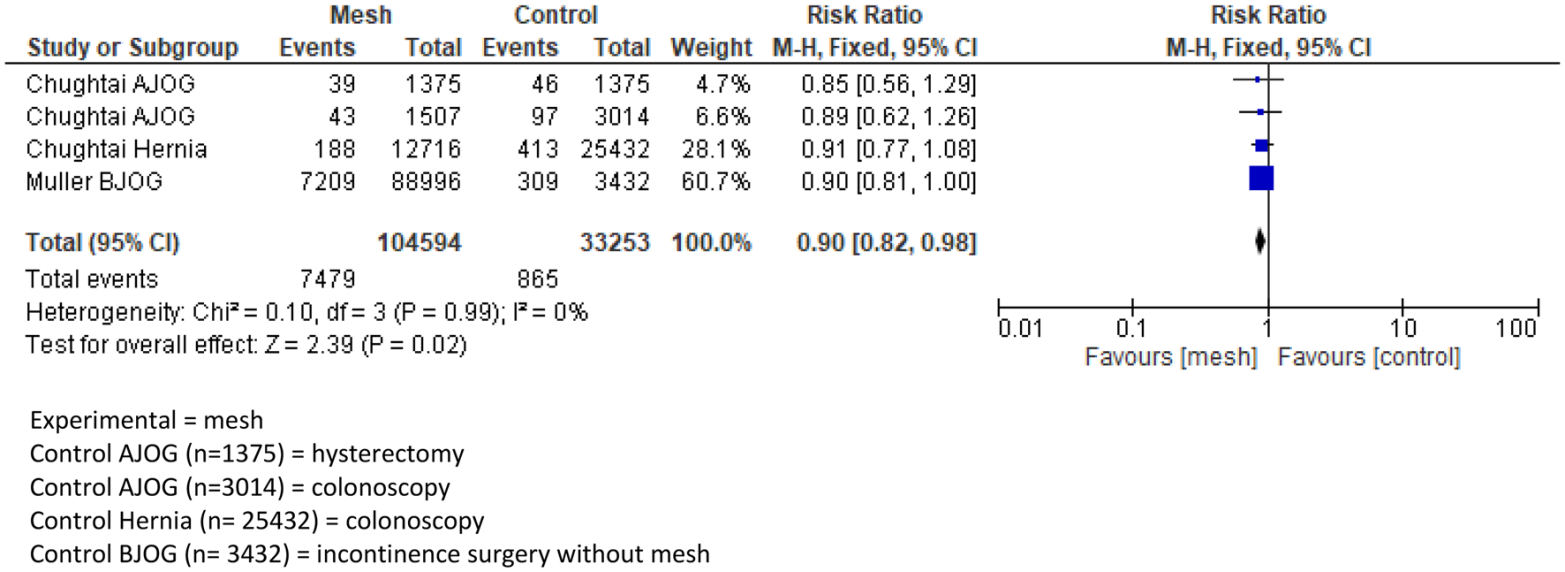
Forest plot of comparison: polypropylene mesh versus no mesh, outcome: systemic autoimmune disorder(s)

A meta-analysis has been performed comparing the outcomes of appropriate studies. The meta-analysis shows no statistically significant association when comparing development of systemic disease after PP implantation and control groups. The calculated risk ratio was 0.9 (95% CI 0.82–0.98) concerning the mesh group, Fig. 2.

## Discussion

In current days, there is a growing concern about the use of PP mesh implants due to mesh-related complications. Some complications have a causal relation with the mesh implants, such as mesh exposure or erosion. In other complications attributed to mesh, such as systemic autoimmune syndromes, this causal relationship remains questionable. The present systematic review aimed at gathering the best scientific evidence currently available regarding the possible association between PP implants for inguinal hernia, ventral hernia or pelvic floor surgery and the development of systemic autoimmune syndromes. The available evidence is scant and should therefore be interpreted with caution. Nonetheless, there appears to be insufficient evidence to conclude an association between PP implants and development of systemic autoimmune syndromes.

The pooled data of Chughtai and Muller et al. showed a RR of systemic autoimmune disorders of 0.9 (95% CI 0.82–0.98) in the PP group. The incidence of systemic autoimmune disorders was 1.5% in the herniorrhaphy mesh group, 2.8% in the POP mesh group and 8.1% in the SUI mesh group. This is comparable with the overall prevalence of autoimmune diseases in the general population, which is estimated to be 3.2–9.4% [15, 16, 20–22]. This is in line with a recent review of Clancy et al. on assessing evidence regarding systemic and autoimmune effects of PP mesh in inguinal hernia repair. The authors found no evidence to link PP with systemic autoimmune syndromes [10].

Thomas et al. performed a review examining the inflammatory response of PP implantation on its host. They found that the inflammatory response persists long after implantation, but no reports were found demonstrating systemic changes due to the implantation of a mesh [23].

Since the available evidence does not show an association between PP mesh implants and the development of systemic autoimmune syndromes, one might wonder why this association has been suggested. This speculation has arisen on consumer websites and discussion platforms, where a multitude of systemic complaints are considered to be related to mesh implants [24, 25].

Mesh implantation triggers a cascade of reactions. The injury at implantation induces a blood–material interaction resulting in provisional matrix formation surrounding the biomaterial [10, 26]. Following this provisional matrix formation, an acute inflammatory response develops. In this phase neutrophil activity is enhanced and histamine and interleukin release from mast cells play an important role [27]. Subsequently, during the chronic inflammatory response, monocytes and lymphocytes can be found surrounding the mesh implant. Finally, there is a foreign-body giant cell formation through fusion of these cells, as they fail to degrade the foreign body [27].

The post-implantation inflammatory responses can elicit an upregulation of systemic inflammatory markers. Systemic levels of CRP and interleukin (IL)-6 are increased in the presence of a mesh [10, 28]. A persistent increased systemic response can theoretically account for the development of autoimmune symptoms [29]. The fact that studies on CRP and IL levels after mesh implantation show that these levels return to normal values within seven days after mesh implantation, however, opposes this theory [10, 28, 30].

Another hypothesis why mesh implants theoretically might be able to cause autoimmune syndromes is that the PP is degraded and absorbed into the systemic circulation [10]. Evidence regarding this possibility is conflicting. Some studies suggest (partial) degradation [31–33], whereas other studies showed no degradation in explanted meshes, up to 14 years after implantation [34, 35].

This review, of course, has its limitations. There are only a few clinical studies regarding this subject. The applied search strategy resulted in only four reports on this topic, even though a comprehensive systematic search has been carried out. It is possible that the small number of studies relevant for this review are attributed to publication bias.

The included studies also had their flaws. Both studies of Chughtai [15, 16] had a minimal risk of bias, but still could have been affected by selection bias. Both studies used the SPARC database for patient selection [19]. This was an administrative database. Clinical data are not available and there is a risk that procedures and diagnosis of autoimmune diseases have been miscoded or missed. Furthermore, all registered inguinal hernia repairs were assumed to have undergone a PP mesh-based repair. This implies that some cases might not have had a PP mesh implant.

The risk of bias of the study of Muller et al. [17] was low, but since an administrative database was used to select patients, this can have inflicted selection bias. Another limitation, as described by the authors is the fact that the data were restricted to hospital admission records. Outpatient data or data of primary care were not included, although this was similar for both the mesh and non-mesh groups.

The study of Cohen Tervaert [9] was limited by the fact that the outcome was present at start of the study. Cases were selected from a population with an alleged autoimmune syndrome (ASIA). The paper described a relatively small size of 40 patients (out of 714) who had PP mesh implanted. Control groups and follow-up were lacking. A diagnostic tool developed for the diagnosis of ASIA has been extrapolated to patients with PP mesh implants. When using a diagnostic test, it should be validated. This diagnostic tool has neither been developed for patients with mesh implants nor has it been validated for this category of patients.

At last, another limitation of the current systematic review involves the partial overlapping of patients in the meta-analysis. The paper of Chughtai involving two control groups and cases were matched with these controls [15]. It remains uncertain, if not likely, that some patients with PP implants were incorporated in both analyses and consequently in the present meta-analysis.

## Conclusion

There is insufficient evidence to conclude that a causal association between PP mesh implants and the development of autoimmune syndromes exists, but consumer websites keep speculating on this association resulting in a lot of patient distress.

We propose a cross-sectional or cohort study measuring the immune status of patients prior to PP mesh implantation. In patients developing systemic complaints, the immune status could be examined again and compared with baseline. This will also enable examination of the potential pathophysiology of systemic complaints. Until such a study has been carried out, physicians should not suggest that PP implants possibly cause autoimmune syndromes. Such suggestions can distress patients, making them ask for operative interventions without a proper indication that can possibly harm them. Instead, physicians should discuss with their patients that the available evidence does not demonstrate a causal association between PP implants and autoimmune syndromes.

## Supplementary Information

Below is the link to the electronic supplementary material.

Supplementary file1 (DOCX 17 KB)

Supplementary file2 (DOCX 18 KB)

## Data Availability

Search strategy and articles are available.
