# Supplementary material for: Are polypropylene mesh implants associated with systemic autoimmune inflammatory syndromes? A systematic review
Source: Hernia. 2022 Jan 12;26(2):401–10. doi: 10.1007/s10029-021-02553-y (PMC9012840; doi:10.1007/s10029-021-02553-y)
Supplement: Supplementary file 1 — Supplementary file1 (DOCX 17 KB) [file 10029_2021_2553_MOESM1_ESM.docx]

**Are polypropylene mesh implants associated with systemic autoimmune inflammatory syndromes? A systematic review.** C.R. Kowalik^1,5^, S.E. Zwolsman, A. Malekzadeh, R.M.H. Roumen , W.A.R. Zwaans ,J.W.P.R. Roovers. *Hernia*

^1^Department of Gynecology, Amsterdam University Medical Centre, The Netherlands

^5^Bergman Clinics, Amsterdam, The Netherlands

c.r.kowalik@amsterdamumc.nl

**Appendix A: Search strategy**

Medline (ovid)

| Database(s): Ovid MEDLINE(R) ALL 1946 to November 22, 2021 | |
| --- | --- |
| # | Searches |
| 1 | exp Pelvic Organ Prolapse/ |
| 2 | exp Uterine Prolapse/ |
| 3 | exp Hernia, Ventral/ |
| 4 | exp Hernia, Inguinal/ |
| 5 | exp Urinary Incontinence, Stress/ |
| 6 | exp Cystocele/ |
| 7 | exp Hernia, Abdominal/ |
| 8 | exp Rectocele/ |
| 9 | exp Herniorrhaphy/ |
| 10 | ((pelvi* or uterin* or uterus or urogenital* or vagin* or inguinal* or groin* or ventral or apical or vagin* vault) adj3 (hernia or prolaps*)).ti,ab,kf. |
| 11 | (colpoplast* or colporrhaph* or hernioplast* or hernioerhaph* or hernia* repair* or prolaps* repair*).ti,ab,kf. |
| 12 | (groin hernia* or inguinal hernia* or hernia inguinal or pelvic prolaps* or pelvic organ prolaps* or urinary stress incontinenc* or ventral hernia* or vagin* hernia or vagin* prolaps* or cystocele or enterocele or rectocele or vaginal vault prolaps* or anterior wall prolaps* or posterior wall prolaps* or middle compartment prolaps*).ti,ab,kf. |
| 13 | 1 or 2 or 3 or 4 or 5 or 6 or 7 or 8 or 9 or 10 or 11 or 12 |
| 14 | exp Polypropylenes/ |
| 15 | Surgical Mesh/ |
| 16 | (propene polymer* or poly-propylen* or propylene polymer* or polypropylen* or hernia* mesh or prolaps* mesh or surgical mesh).ti,ab,kf. |
| 17 | ((Gynemesh or Coloplast or Restorelle or apogee perigee or Elevate or avaulta or prolift, IVS or MiniArc or Altis or Ajust, RetroArc or Bard Align) adj5 (poly-propylene* or polypropylen* or POP or mesh or vagin* repair* or prolaps* repair* or vagin* surg* or pelvic* surg*)).ti,ab,kw. |
| 18 | 14 or 15 or 16 or 17 |
| 19 | exp Autoimmunity/ |
| 20 | exp Autoimmune Diseases/ |
| 21 | exp Systemic Inflammatory Response Syndrome/ |
| 22 | Inflammation/ |
| 23 | exp Foreign-Body Reaction/ |
| 24 | ((chronic* or systemic or persist*) adj3 (inflammat* or immun*)).ti,ab,kf. |
| 25 | ((immun* or inflammat* or autoimmun* or auto-immun*) adj3 (respons* or activat* or syndrome)).ti,ab,kf. |
| 26 | (autoimmun* or auto-immun* or autoinflam* or auto-inflam* or systemic inflam* or foreign body reaction or chronic* inflamm* or ASIA syndrome).ti,ab,kf. |
| 27 | 19 or 20 or 21 or 22 or 23 or 24 or 25 or 26 |
| 28 | 13 and 18 and 27 |

Embase (ovid)

| Database(s): Ovid MEDLINE(R) ALL 1946 to November 22, 2021 | |
| --- | --- |
| # | Searches |
| 1 | exp Pelvic Organ Prolapse/ |
| 2 | exp Uterine Prolapse/ |
| 3 | exp Hernia, Ventral/ |
| 4 | exp Hernia, Inguinal/ |
| 5 | exp Urinary Incontinence, Stress/ |
| 6 | exp Cystocele/ |
| 7 | exp Hernia, Abdominal/ |
| 8 | exp Rectocele/ |
| 9 | exp Herniorrhaphy/ |
| 10 | ((pelvi* or uterin* or uterus or urogenital* or vagin* or inguinal* or groin* or ventral or apical or vagin* vault) adj3 (hernia or prolaps*)).ti,ab,kf. |
| 11 | (colpoplast* or colporrhaph* or hernioplast* or hernioerhaph* or hernia* repair* or prolaps* repair*).ti,ab,kf. |
| 12 | (groin hernia* or inguinal hernia* or hernia inguinal or pelvic prolaps* or pelvic organ prolaps* or urinary stress incontinenc* or ventral hernia* or vagin* hernia or vagin* prolaps* or cystocele or enterocele or rectocele or vaginal vault prolaps* or anterior wall prolaps* or posterior wall prolaps* or middle compartment prolaps*).ti,ab,kf. |
| 13 | 1 or 2 or 3 or 4 or 5 or 6 or 7 or 8 or 9 or 10 or 11 or 12 |
| 14 | exp Polypropylenes/ |
| 15 | Surgical Mesh/ |
| 16 | (propene polymer* or poly-propylen* or propylene polymer* or polypropylen* or hernia* mesh or prolaps* mesh or surgical mesh).ti,ab,kf. |
| 17 | ((Gynemesh or Coloplast or Restorelle or apogee perigee or Elevate or avaulta or prolift, IVS or MiniArc or Altis or Ajust, RetroArc or Bard Align) adj5 (poly-propylene* or polypropylen* or POP or mesh or vagin* repair* or prolaps* repair* or vagin* surg* or pelvic* surg*)).ti,ab,kw. |
| 18 | 14 or 15 or 16 or 17 |
| 19 | exp Autoimmunity/ |
| 20 | exp Autoimmune Diseases/ |
| 21 | exp Systemic Inflammatory Response Syndrome/ |
| 22 | Inflammation/ |
| 23 | exp Foreign-Body Reaction/ |
| 24 | ((chronic* or systemic or persist*) adj3 (inflammat* or immun*)).ti,ab,kf. |
| 25 | ((immun* or inflammat* or autoimmun* or auto-immun*) adj3 (respons* or activat* or syndrome)).ti,ab,kf. |
| 26 | (autoimmun* or auto-immun* or autoinflam* or auto-inflam* or systemic inflam* or foreign body reaction or chronic* inflamm* or ASIA syndrome).ti,ab,kf. |
| 27 | 19 or 20 or 21 or 22 or 23 or 24 or 25 or 26 |
| 28 | 13 and 18 and 27 |

Scopus

TITLE-ABS-KEY ( "pelvic organ Prolaps*" OR "uterine Prolaps*" OR "ventral hernia*" OR "inguinal hernia*" OR "urinary stress incontinenc*" OR cystocele OR rectocele OR enterocele OR "vaginal vault prolaps*" OR "apical prolaps*" OR "anterior wall prolapse*" OR "posterior wall prolaps*" OR "middle compartment prolaps*" OR herniorrhaphy OR colporrhaphy ) OR ( TITLE-ABS-KEY ( pelvi* OR uterin* OR uterus OR urogenital* OR vagin* OR inguinal* OR groin* OR ventral OR apical OR "vagin* vault" ) W/3 ( hernia OR prolaps* ) ) AND TITLE-ABS-KEY ( "propene polymer*" OR "poly-propylen*" OR "propylene polymer*" OR polypropylen* OR "hernia* mesh" OR "prolaps* mesh" OR "surgical mesh" ) OR TITLE-ABS-KEY ( gynemesh OR coloplast OR restorelle OR "apogee perigee" OR elevate OR avaulta OR prolift OR ivs OR miniarc OR altis OR ajust OR retroarc OR "Bard Align" ) W/3 ( "poly-propylene*" OR polypropylen* OR pop OR mesh OR "vagin* repair*" OR "prolaps* repair*" OR "vagin* surg*" OR "pelvic* surg*" ) AND TITLE-ABS-KEY(Autoimmunity or "autoimmune Diseas*" or "Systemic Inflammatory Response Syndrome" or "inflammation" or "Foreign-Body Reaction" or "Foreign Body Reaction" or "chronic* inflamm*" or "ASIA syndrome") OR TITLE-ABS-KEY((chronic* or systemic or persist*) W/3 (inflammat* or immun*))

Web of science

| #1 | TS=("pelvic organ Prolaps*" or "uterine Prolaps*" or "ventral hernia*" or "inguinal hernia*" or "urinary stress incontinenc*" or cystocele or rectocele or enterocele or "vaginal vault prolaps*" or "apical prolaps*" or "anterior wall prolapse*" or "posterior wall prolaps*" or "middle compartment prolaps*" or herniorrhaphy or colporrhaphy ) |
| --- | --- |
| #2 | TI=("pelvic organ Prolaps*" or "uterine Prolaps*" or "ventral hernia*" or "inguinal hernia*" or "urinary stress incontinenc*" or cystocele or rectocele or enterocele or "vaginal vault prolaps*" or "apical prolaps*" or "anterior wall prolapse*" or "posterior wall prolaps*" or "middle compartment prolaps*" or herniorrhaphy or colporrhaphy) or AB=("pelvic organ Prolaps*" or "uterine Prolaps*" or "ventral hernia*" or "inguinal hernia*" or "urinary stress incontinenc*" or cystocele or rectocele or enterocele or "vaginal vault prolaps*" or "apical prolaps*" or "anterior wall prolapse*" or "posterior wall prolaps*" or "middle compartment prolaps*" or herniorrhaphy or colporrhaphy) |
| #3 | AB=((pelvi* or uterin* or uterus or urogenital* or vagin* or inguinal* or groin* or ventral or apical or "vagin* vault") near/3 (hernia or prolaps*) ) |
| #4 | ((#3) OR #2) OR #1 |
| #5 | TS=(Polypropylenes or "Surgical Mesh") |
| #6 | TI=(“propene polymer*” or “poly-propylen*” or “propylene polymer*” or polypropylen* or “hernia* mesh” or “prolaps* mesh” or “surgical mesh”) or AB=(“propene polymer*” or “poly-propylen*” or “propylene polymer*” or polypropylen* or “hernia* mesh” or “prolaps* mesh” or “surgical mesh”) |
| #7 | AB=((Gynemesh or Coloplast or Restorelle or "apogee perigee" or Elevate or avaulta or prolift or IVS or MiniArc or Altis or Ajust or RetroArc or "Bard Align") near/3 ("poly-propylene*" or polypropylen* or POP or mesh or "vagin* repair*" or "prolaps* repair*" or "vagin* surg*" or "pelvic* surg*") ) |
| #8 | ((#5) OR #6) OR #7 |
| #9 | TS=(Autoimmunity or "autoimmune Diseas*" or "Systemic Inflammatory Response Syndrome" or "inflammation" or "Foreign-Body Reaction" or "Foreign Body Reaction" or "chronic* inflamm*" or "ASIA syndrome") |
| #10 | TI=((immun* or inflammat*) near/3 (respons* or activat* or syndrome*) ) or AB=((immun* or inflammat*) near/3 (respons* or activat* or syndrome*) ) |
| #11 | TI=(Autoimmunity or "autoimmune Diseas*" or "Systemic Inflammatory Response Syndrome" or "inflammation" or "Foreign-Body Reaction" or "Foreign Body Reaction" or "chronic* inflamm*" or "ASIA syndrome") or AB=(Autoimmunity or "autoimmune Diseas*" or "Systemic Inflammatory Response Syndrome" or "inflammation" or "Foreign-Body Reaction" or "Foreign Body Reaction" or "chronic* inflamm*" or "ASIA syndrome") |
| #12 | ((#9) OR #10) OR #11 |
| #13 | ((#4) AND #8) AND #12 |
