# Supplementary material for: Are polypropylene mesh implants associated with systemic autoimmune inflammatory syndromes? A systematic review
Source: Hernia. 2022 Jan 12;26(2):401–10. doi: 10.1007/s10029-021-02553-y (PMC9012840; doi:10.1007/s10029-021-02553-y)
Supplement: Supplementary file 2 — Supplementary file2 (DOCX 18 KB) [file 10029_2021_2553_MOESM2_ESM.docx]

| Major Criteria:  • Exposure to an external stimuli (Infection, vaccine, silicone, adjuvant) prior to clinical manifestations.  • The appearance of ’typical’ clinical manifestations:   – Myalgia, Myositis or muscle weakness   – Arthralgia and/or arthritis   – Chronic fatigue, un-refreshing sleep or sleep disturbances   – Neurological manifestations (especially associated with demyelination)   – Cognitive impairment, memory loss   – Pyrexia, dry mouth  • Removal of inciting agent induces improvement  • Typical biopsy of involved organs |
| --- |
| Minor Criteria:  • The appearance of autoantibodies or antibodies directed at the suspected adjuvant  • Other clinical manifestations (i.e. irritable bowel syn.)  • Specific HLA (i.e. HLA DRB1, HLA DQB1)  • Evolvement of an autoimmune disease (i.e. MS, SSc) |
